# Supplementary material for: Diverging effects of host density and richness across biological scales drive diversity-disease outcomes
Source: Nat Commun. 2024 Mar 2;15:1937. doi: 10.1038/s41467-024-46091-4 (PMC10908850; doi:10.1038/s41467-024-46091-4)

SUPPLEMENTARY INFORMATION

Pond sampling

To understand interactions between amphibians and their trematode parasite communities, we sampled 224 ponds distributed across the Bay Area of California (Santa Clara, Alameda, and Contra Costa counties) between 2009 and 2019. Each pond was sampled twice during a given year (early and late summer), and often in multiple years (average number of years in which a pond was sampled = 3.47 ± 0.24 SE). During the 'initial conditions' visit in early summer (May to June), we surveyed the aquatic community using a combination of dipnet and seine surveys[1]. For dipnet surveys, we used a D-frame dipnet (1.4 mm mesh, 2600 cm^2 opening) to conduct sweeps every 3 to 5 m around the pond perimeter. Each sweep was 1 m in length, beginning from offshore and moving directly toward the shoreline in a rapid movement through water and vegetation. Contents of each sweep were transferred to a sorting tray in which taxa were identified to lowest taxonomic level and counted. Amphibian larvae were identified to species, snails to species or genus, and other invertebrates to family. Based on previous experimental studies[2-4], we focused on larval damselflies (suborder Zygoptera) as key potential predators of trematode cercariae. This included members of the families Coenagrionidae (e.g., *Enallagma* spp.) and Lestidae (e.g., *Lestes* spp.). The density of each taxon was estimated as their average number per dipnet sweep. We supplemented dipnet surveys with three to five habitat-stratified seine hauls (4 mm mesh, 1 m tall by 2 m wide) conducted haphazardly from the deeper part of the pond toward shore. Seines enhanced detection for some difficult to capture species, such as larval bullfrogs (*Rana catesbeiana*) and larval tiger salamander (*Ambystoma californiense*)[5].

During the 'transmission assessment' visit in late June through August (depending on the pond hydroperiod), we collected 10-15 late-stage amphibian larvae or recently metamorphosed individuals from all non-threatened host species for dissection, including *Pseudacris regilla* (Pacific chorus frog), *Anayxrus boreas* (Western toad), *Rana catesbeiana* (American bullfrog), *Taricha torosa* (California newt), and *T. granulosa* (rough-skinned newt). We focused on animals at or nearing metamorphosis to provide a standardized life stage for quantifying water-borne infections acquired during larval development. For bullfrogs, which tend to emerge later than other anurans and can be challenging to capture in adequate numbers, we conducted additional, night-time visits with the aid of a headlamp to enhance capture success. Sampling equipment was rigorously decontaminated between sites to avoid inadvertent spread of free-living or pathogenic organisms.

Dissections of amphibian and snail hosts

All major organs and tissues (e.g., heart, liver, intestines, rectum, stomach, tongue, mandible, kidneys, and fat bodies) were examined for macroparasites under an Olympus SZX10 stereodissecting microscope[6-8]. Dissections were performed on fresh specimens to enhance our ability to identify relevant morphological features of detected parasites. We closely inspected the body cavity, muscles, and skin for encysted trematodes. Isolated live parasites were counted and identified to the lowest taxonomic level based on morphological traits[9-13]. We placed particular emphasis on the larval stages (metacercaria or mesocercaria) of four digenetic trematode taxa: *Ribeiroia ondatrae* (hereafter "*Ribeiroia*"), *Alaria marcinae* (hereafter "*Alaria*"), *Cephalogonimus americanus* (hereafter "*Cephalogonimus*"), and *Echinostoma* spp. (echinostome infections in amphibians likely represent a mixture of *Echinostoma*/*Echinoparyphium*) (hereafter "*Echinostoma*"). These four parasite taxa comprise 95% of observed macroparasitic infections in juvenile amphibians[5] and each use *Helisoma trivolvis* snails as first intermediate hosts (*Echinostoma* can also use *Physa* spp.). For snails, dissections of ~100 individuals per species were performed by crushing the outer shell and carefully teasing apart the soft tissues to detect trematode stages (sporocysts, rediae, and/or

cercariae). Snail infections were identified based on characteristics of the specific cercariae morphotype[11] and complementary genetic sampling. Immature (prepatent) infections without identifiable cercariae were not included in estimates of infection prevalence. Because very young snails are both harder to quantify and less likely to support patent trematode infections, we dissected only snails above a minimum size threshold (5 mm for *Helisoma trivolvis* and 1 mm for *Physa* spp.).

Formulation of predictor variables

<u>*Infection pressure*</u>: To estimate amphibian exposure to infective cercariae, we developed three alternative metrics representing 'infection pressure': prevalence of infection in snails, density of infected snails, and estimated density of infective cercariae. Prevalence of infection represents the number of infected snails divided by the number dissected. Density of infected snails was estimated as the product of snail density (average number captured per dipnet sweep) and the prevalence of snails exhibiting infection. Our estimate of cercaria densities included two additional pieces of information. Specifically, we multiplied the average size of dissected snails in a pond by a regression equation relating snail size to cercariae release over 24 hours (as quantified from a subset of infected snails for each trematode under standardized conditions, see Table S6). This relationship recognizes that the number of cercariae emerging from infected snails varies substantially as a function of snail size[14]. The product of terms – snail density, infection prevalence, mean size and the size-specific slope of cercariae release – was used to generate estimates of cercaria density for each trematode species and site-by-year observation. We determined which of these estimates served as the best proxy for exposure by regressing them against observed infections in *P. regilla* (our indicator species) and comparing model fit with AIC. These models took the same form as transmission models at the individual host scale, but without any factors representing hypotheses related to diversity. With the function glmmTMB[15], we ran generalized linear mixed effects models (overdispersed Poisson distribution) with total metacercariae observed in a pond as the response variable and an offset term for the number of amphibians dissected. Our estimate of infection pressure was the only fixed effect, and we included random effects for site, year, and an observation-level random effect for the site-year combination. These preliminary analyses indicated that the estimated density of infective cercariae was by far the best predictor of infection success in *P. regilla* (Table S7 and Fig. S1), and we therefore used this proxy to represent "infection pressure" in all subsequent models.

Because trematode infective stages within snails were not always detected, even in cases for which amphibians were found infected and >100 snails were dissected, we imputed a low value of infection prevalence (0.5%) to snail populations for which infections were recorded in amphibian second intermediate hosts but not detected among examined snails. This assumption stems from the fact that infected snails must be present in order for amphibian larvae and recent metamorphs to host infections. The addition of this small, non-zero value helped to address what would otherwise be a lack of variance in infection pressure by preventing the product of the constituent terms from multiplying out to zero. Given that amphibian infections by echinostome trematodes can come from *H. trivolvis* or *Physa* spp. snails, we created a joint infection pressure term based on the sum of their individual values for sites at which both species were present and dissected. Values of infection pressure were $\log_{10}$-transformed (+1) prior to incorporation in our main (hypothesis-testing) models as an 'a priori obvious' predictor term. Thus, while we expected infection pressure to positively predict infection success in amphibians for each trematode species, our primary interest was in variables that altered the slope of this relationship (i.e., significant interaction terms between infection pressure and additional covariates).

<u>*Host richness and density*</u>: we calculated the richness of larval amphibian hosts using species detections derived from dipnet surveys as well as other collection methods. This

recognized the fact that some species can be difficult to detect through dipnet surveys alone, particularly when at low density. Because amphibian communities with richness values of five species or greater were relatively rare in the dataset (10 of 549 records), we combined them into a species richness of '4 or greater' for the purposes of analyses. Host density was estimated using the average number of detected larvae per dipnet sweep. For total host density at the pond level, we summed the dipnet density values of all non-threatened host species to provide an overall value of larval amphibian density. Including threatened species – which lacked information on parasite infection because they could not be dissected – would systematically underestimate infection (although analyzing the data while excluding all ponds that supported threatened species did not appreciably change results).

Statistical analysis

To evaluate relationships between diversity and parasite transmission, we used generalized linear mixed models (GLMM) because (1) they allow for Poisson and negative binomial distributions that are more likely to approximate aggregated parasite counts[16-18], and (2) infection data from host individuals or species can be nested within ponds using random intercept terms[19,20]. Our general approach was to test how infection pressure and its interactions with variables representing host density, host richness, and predator density affected infection success in amphibians, either at the biological scale of individual hosts or aggregated among all amphibian species in the community (see Fig. 1 and *Glossary*). Note that we use biological scale here to refer to the level of biological organization, rather than to spatial grain or extent, for which there is a separate literature focused on the diversity-disease relationship[21,22]. We used the Anova function in the R package 'car'[23] to assess the overall significance of interactions.

Individual scale (the host perspective): For each trematode species, we constructed models to evaluate what factors influenced infection success in individuals (measured as the average number of metacercariae per individual host). In the models, the response variable was the total number of metacercariae quantified among all hosts of a given species in a given pond, with an offset used to account for the number of dissected hosts. We modeled metacercaria counts as an overdispersed Poisson distribution by including an observation-level random effect to account for aggregation (although we also explored using a negative binomial distribution with or without zero inflation, with similar results). Pond identity and year of sampling were included as random intercept terms to account for likely sources of autocorrelation. As fixed effects, all models included terms for infection pressure as well as main effects for host richness, host density, and predator density. Interactions between infection pressure and each term were included to assess whether such variables altered the slope of the relationship between infection pressure and observed infection success in amphibians (i.e., as a metric of transmission between infected snails and suitable amphibian hosts). We initially also considered the potential role of pond area, which could influence the contact rate between hosts and infective parasites, but this term was non-influential and therefore removed from subsequent models. To focus on testing what variables affected parasite transmission from snails to amphibians (rather than colonization into a pond), we only included ponds in which a given parasite was detected within a host (either in snails or amphibians). We further limited observations to cases in which ponds were <300 m in perimeter, at least 20 snails were dissected, at least 5 amphibians of a given species were dissected, and amphibian larvae were detected within dipnet surveys. Sites without snails or amphibians were omitted. Data were collected between 2009 and 2019, although for analyses of *Echinostoma* we restricted the data to years of 2011 and later when we dissected both *Helisoma trivolvis* and *Physa* spp.

Community scale (the parasite perspective): We conducted a second set of analyses targeted at the community scale, for which infection success was measured as the total density of established metacercariae summed across all hosts in the same pond. Evaluating this question is essential for understanding how net parasite transmission varies among different

host communities; for instance, even if increases in host diversity reduce average infection load in a particular host species, this may have no effect on net transmission if infections increase concurrently in other host species. Similarly, if total host density increases with host richness (i.e., additive assembly), richer communities may support a greater total density of parasites, even if at a lower average infection load per host. We again tested how our diversity-linked mechanisms, host richness, total host density, and aquatic predators, interacted with infection pressure to determine the total density of successfully established metacercariae, quantified as the sum of each host species' infection load (average metacercariae per host, from dissections during the transmission assessment visit) multiplied by its larval density (hosts per netsweep, collected during the initial conditions visit) (see Fig. 1). Because this quantity was continuous rather than discrete, we modeled it as a Gaussian response after $\log_{10}$-transformation (+1). Pond identity and sample year were incorporated as random intercept terms, and we only included site-year combinations in which host species detected in netsweep surveys also had assessments of infection. These analyses asked how core community structure factors—total host density, the richness and composition of amphibian hosts, and the density of potential predators—influenced parasite infection success across the entire amphibian community, rather than average infection load in specific host individuals or species.

As with the individual scale analysis, we only included ponds in which a given parasite was detected, ponds <300 m in perimeter, site-years in which at least 20 snails and at least 5 amphibians of any species were dissected, and amphibian larvae were detected within dipnet surveys. Sites in which no amphibian species were detected were omitted. Data were collected between 2009 and 2019, although for analyses of *Echinostoma* we restricted the data to 2011 and later when we dissected both *Helisoma trivolvis* and *Physa* spp. To ensure alignment between the dipnet survey data and the dissections, we only included sites for which each amphibian species detected in dipnets also had corresponding dissection data. If a species was dissected but not detected in the dipnets, these data were still included (but presumably had no influence on infection loads since densities were effectively zero). As such, all sites with threatened species – which could not be dissected – were omitted (although including these sites did not appreciably alter our results). This helped ensure that we did not underestimate the total number of parasites by including sites for which not all host species could be dissected. We repeated these analyses for each of the four trematode parasites.

Comparing across biological scales: Our focus on parasite transmission can be addressed with two separate scales: *i)* the individual scale ('host perspective'), which considers how many parasites an individual is infected by, and *ii)* the community scale ('parasite perspective'), which considers a parasite's probability of successfully infecting a host (Fig. 1). Theory predicts that these separate scales can yield different diversity-disease relationships[24], so we overlaid these two scales to explore this possibility. Infection success in individuals (the host perspective) was represented with log-transformed metacercariae per host for all host species, which we plotted using each parasite's individual scale model. Because these models contained richness as a significant effect, we used the predict function in R[25] to generate model-estimated numbers of metacercariae per host for each richness level. Infection success in the community (the parasite perspective) was represented with log-transformed metacercariae density (summing the metacercariae densities across amphibian host species), which we plotted using each parasite's community-scale transmission model. The community-scale transmission models contained community competence as a predictor, which is a composite of species' presences, densities, and lab-derived competence values (i.e., for each community competence value, there is an associated richness value). After using the predict function to generate model-estimated metacercaria densities for each community competence level, we appended these estimates to their corresponding richness levels. For model predictions at both scales (e.g., Figs. 4 and 5), we had to select a constant infection pressure (because infection pressure was a core variable in all transmission models). We generated predictions at high

infection pressures (log_{10}-transformed cercariae density = 2) to simulate a high-risk environment. In generating the model predictions, all other fixed effects were held at their mean values, while excluding random effects.

Outlier treatment and model diagnostics

The presence and potential influence of outliers were explored using three methods. First, we generated plots of the relationship between infection pressure (from snail intermediate hosts) and observed infection success in *P. regilla* (our indicator species) for each trematode, with estimated cercariae density on the x-axis and mean metacercariae per *P. regilla* on the y-axis (both variables were log_{10} + 1 transformed). Any observations for which infection pressure was high (>1.5) and amphibian infection approached zero were considered potential outliers and were further examined or removed. Second, we investigated residuals from the fitted relationship between infection pressure and *P. regilla* infections, for which potential outliers were considered as observations whose residuals were more than 3 standard deviations greater than the mean residual. Third, we calculated Cook's distance from these models (using the package influence.ME[26]), where potential outliers were considered those whose distance was greater than 4/N. Any observations that were identified as potential outliers using at least two of the three methods were removed, leading to the removal of 4, 4, 4 and 2 site-by-year observations *for Alaria*, *Cephalogonimus*, *Echinostoma*, and *Ribeiroia*. This represented between 0.4% and 1.6% of observations for each parasite.

For model diagnostics, we used the performance package[27] to calculate the marginal and conditional R^2 values, estimate variance inflation factors for predictors (as an assessment of collinearity among predictor variables), test for overdispersion, and inspect normality of the random effects.

Sampling overview

Across 11 years of sampling, we surveyed 224 sites, 1,918 amphibian host populations (site x year x host species), and 17,317 amphibian hosts for infection. On average, each site (pond) was sampled for 3.47 ± 0.24 SE years. Among sites with at least one amphibian species, amphibian species richness ranged from 1 to 6 with a mean ± 1 SE of 2.55 ± 0.031 (n = 873). *Pseudacris regilla* was the most commonly encountered amphibian species, occurring in 95% of site-years, followed by *T. torosa* (76%), *A. boreas* (38%), *T. granulosa* (24%), *R. catesbeiana* (16%), *R. draytonii* (8%), and *A. californiense* (2%). With respect to the four major trematode taxa, *Echinostoma* was both the most frequently observed (72.5% of 871 site-years) and exhibited the highest average loads in amphibians when present (44.63 ± 4.85; n = 931 populations from 655 site-years), followed by *Ribeiroia* (49.7% and 8.85 ± 1.05; n = 710 populations from 433 site-years), *Cephalogonimus* (25.9% and 7.69 ± 2.69; n = 266 populations from 226 site-years), and *Alaria* (20.3% and 9.66 ± 1.58; n = 224 populations from 177 site-years).

To characterize infection pressure stemming from first intermediate hosts, we measured and quantified the infection status of 218,930 aquatic snails, including 135,591 *Helisoma trivolvis* and 83,339 *Physa* spp. Both snail taxa were common, with *Helisoma trivolvis* occurring in 75.6% of site-years and *Physa* spp. in 64%. Infection prevalence ranged from 0 to 87.5%, with an overall average of 11.1% for *H. trivolvis* (n = 607 site-years) and 8.8% for *Physa* spp. (n = 387 site-years) (*Physa* were only examined for infection between 2011 and 2019). With respect to frequency of detections, *Echinostoma* was the most commonly encountered trematode infection, present in 50% of site-years with *H. trivolvis* and 74.6% of site-years with *Physa* spp., followed by *Alaria* (44.3% of site-years; longifurcate-apharyngeate cercaria), *Ribeiroia* (31% site-years; gymnocephalous cercaria), and *Cephalogonimus* (25.5% of site-years; armatae xiphidocercaria). For further information on cercaria morphotypes, see Schell (1985).

Infection success at the individual scale

Infection pressure (a proxy for cercaria density from infected snails) was a strong, positive predictor of observed infections in *P. regilla*. The slope of this relationship was strongest for *Alaria* (coefficient = 3.31; $P < 0.00001$) followed by *Cephalogonimus* (coefficient = 2.25; $P < 0.00001$), *Ribeiroia* (coefficient = 2.18; $P < 0.00001$) and *Echinostoma* (coefficient = 0.96; $P < 0.00001$) (Fig. 2; Table S1). The interaction between infection pressure and host richness was significant and negative in the models for all four parasite species (Table S1), indicating that parasite infection success in individuals decreased with increasing host richness. The density of focal host (*P. regilla*) larvae manifested as a positive main effect for *Cephalogonimus* (coefficient = 0.488; $P = 0.012$) and as a positive interaction with pressure for *Ribeiroia* (coefficient = 0.25; $P = 0.038$), but was uninfluential for other parasites. The density of predators, either as a main effect or an interaction with pressure, had little influence on transmission. The marginal R^2 values ranged from 0.20 (*Echinostoma*) to 0.37 (*Alaria*) (Table S1), and there was no evidence of collinearity (all variance inflation factors < 1.5; correlation matrices of predictors available in Table S8).

When all host species were incorporated into a model simultaneously, host richness continued to exhibit a negative influence on infection success in individuals, via an interaction with infection pressure (Tables S2 & S3). For *Ribeiroia*, there was an additional 3-way interaction among infection pressure, host richness, and amphibian species identity (comparison of model with and without the 3-way interaction: chi-square = 12.699; df = 4; $P = 0.013$). More specifically, the protective influence of richness was greatest for bullfrogs (*R. catesbeiana*) (infection pressure x richness x bullfrog interaction coefficient = 0.37; $P = 0.032$; see Fig. 3). For each of the other three parasites, host richness broadly decreased infections for all host species, with no such 3-way interaction detected (Tables S2 & S3). The 3-way interaction was therefore removed for these parasites and the models re-run. The marginal R^2 values ranged from 0.29 (*Ribeiroia*) to 0.52 (*Echinostoma*) (Table S3), and there was little evidence of collinearity (the VIF for the 3-way interaction in the *Ribeiroia* model was 2.7; all other variance inflation factors were < 1.12).

The analysis further highlighted dramatic differences in infections among amphibian species, even when controlling for sample site and sample year. For *Echinostoma*, the greatest infection loads were observed in *P. regilla* and *R. catesbeiana*, with lower loads in *A. boreas* and the lowest values in the two newt species (*T. torosa* and *T. granulosa*) (Table S9). For *Ribeiroia*, *A. boreas* supported the lowest average loads while *T. torosa* and *R. catesbeiana* had the highest, with intermediate values for *P. regilla* and *T. granulosa*. For *Alaria*, *P. regilla* had higher loads than *A. boreas* and *R. catesbeiana*, while the two newts had infection values of zero or approaching zero. For *Cephalogonimus*, *P. regilla* had the highest infections, followed by *A. boreas*, *T. granulosa*, and then *R. catesbeiana*, with very few infections detected in *T. torosa* (Table S9).

Infection success at the community scale

After accounting for host density, increases in host richness also led to progressive declines in the infection success of cercariae across the amphibian community (Table S4). Thus, the infection pressure-by-richness interaction was a negative predictor in the models for three of the four parasites. For *Echinostoma*, the coefficient for infection pressure-by-richness was negative but not significant (coefficient = -0.063; $P = 0.0135$) (Table S4). Total host density, summed across all host species at the same site, had a positive main effect and a positive interaction with infection pressure for three of the four parasites. Predators did not have influential effects on transmission. For *Cephalogonimus* only, predator density had a positive (rather than negative) main effect on infection success and a positive interaction with infection pressure (Table S4). Predictors were not collinear (Table S8).

As an alternative to using specific terms for host richness and host density, , we also tested models that instead included community competence (i.e., the density-weighted competence value for the amphibian community, which integrated information on host identity, density, and lab-derived competence values). The AIC values of these models were compared against a model that included terms for infection pressure, host richness, host density (summed among all host species in the same pond), and two-way interaction terms between infection pressure and each of the other terms. Based on AIC, the community competence models were generally superior to the previously described models, with ΔAIC values of 16 to 25 lower (see Table S5). For *Ribeiroia*, the competence model was within 5.8 AIC units of the alternative model. The coefficients for community competence and its interaction with infection pressure were generally positive, indicating that as infection pressure increased, parasite infection success was highest among dense amphibian communities with high competence. The relative importance of host competence and host density roughly mirrored the degree of host specificity for a given parasite. Thus, the coefficient for the pressure-by-community competence interaction was greatest for *Alaria* and *Cephalogonimus*, which also exhibited the greatest degree of variance in infection success among host species (as an indicator of specificity), whereas this coefficient was more moderate for *Echinostoma* and *Ribeiroia*, which were broadly successful in infecting different amphibian species (as reflected by a lower variance in infection success among host species in the field as well as experimental trials).

Experimental competence trials

Competence values for each host-parasite interaction were obtained experimentally via a series of exposure trials[28]. In brief, each host species was exposed to a range of cercaria doses for each parasite species. The range of doses (0, 20, 40, 100, and 200 cercariae per host) was selected to replicate an exposure gradient that hosts are likely to experience in the wild. With this experimental design, dose-response curves were generated for host susceptibility (likelihood of infection) and host survival (likelihood the host would survive to permit downstream transmission), where the product of these two curves represents a host's competence as a function of dose. By integrating the area under the competence curve for each host-parasite interaction, we arrived at a standard value of competence for the pairings. Competence values for each of the four parasites were scaled between 0 and 1 (where 1 represents the maximum observed competence value; see Table S10).

Relationship between host density and host richness

We explored how host density and richness covary by running simple models exploring amphibian species richness (of non-endangered species) as the predictor variable and total amphibian density as the response variable (summed density of all non-endangered amphibians per dipnet sweep) with each community observed at a given site in a given year (a "site-year") as the unit of replication. We explored two model forms: a linear model using the 'lm' function in R, and a saturating function using the 'nls' function in R and following the Michaelis-Menten equation. For both models, we included an intercept of zero because of the implicit assumption that the curve will go through the origin (ponds with no amphibians will have zero richness and zero density). We then compared the model fits with AIC. While models were run on the raw density values (left panel of Fig. S2), we also plot the data and model predictions following $\log_{10}(+1)$ transformation for ease of visualization (right panel of Fig. S2). The linear model (black line) and saturating model (red line) had nearly identical fits to the data (linear AIC: 4853.88, saturating AIC: 4853.834), suggesting that the saturating function does not improve model fit. The increase of total host density with host richness suggests that these communities are additive, leading to increased host supply (a resource for parasites) at higher richness levels.

References

1 Johnson, P. T. J., Preston, D. L., Hoverman, J. T. & Richgels, K. L. Biodiversity decreases disease through predictable changes in host community competence. *Nature* **494**, 230-233 (2013).

2 Orlofske, S. A., Jadin, R. C., Preston, D. L. & Johnson, P. T. J. Parasite transmission in complex communities: predators and alternative hosts alter pathogenic infections in amphibians. *Ecol.* **93**, 1247-1253 (2012).

3 Orlofske, S. A., Jadin, R. C. & Johnson, P. T. J. It's a predator–eat–parasite world: how characteristics of predator, parasite and environment affect consumption. *Oecologia* **178**, 537-547 (2015).

4 McDevitt-Galles, T., Carpenter, S. A., Koprivnikar, J. & Johnson, P. T. J. How predator and parasite size interact to determine consumption of infectious stages. *Oecologia* **197**, 551-564 (2021).

5 Moss, W. E., McDevitt-Galles, T., Calhoun, D. M. & Johnson, P. T. J. Tracking the assembly of nested parasite communities: using β-diversity to understand variation in parasite richness and composition over time and scale. *J. Anim. Ecol.* **89**, 1532-1542 (2020).

6 Hartson, R. B., Orlofske, S. A., Melin, V. E., Dillon, R. T. & Johnson, P. T. J. Land use and wetland spatial position jointly determine amphibian parasite communities. *EcoHealth* **8**, 485-500 (2011).

7 Johnson, P. T. J. *et al.* Habitat heterogeneity drives the host-diversity-begets-parasite-diversity relationship: evidence from experimental and field studies. *Ecol. Lett.* **19**, 752-761 (2016).

8 Calhoun, D. M. *et al.* Patterns of *Clinostomum marginatum* infection in fishes and amphibians: integration of field, genetic, and experimental approaches. *J. Helminthol.* **94**, 1-12 (2019).

9 Smyth, J. D. & Smyth, M. M. *Frogs as Host-Parasite Systems 1*. (Macmillan Press Ltd., 1980).

10 Prudhoe, S., Bray, R. A. *Platyhelminth Parasites of the Amphibia*. (British Museum of Natural History, 1982).

11 Schell, S. C. *Handbook of Trematodes of North America North of Mexico*. (University Press of Idaho, 1985).

12 Sleigh, M. A. *Protozoa and Other Protists*. (CUP Archive, 1991).

13 Gibson, D. I., Jones, A. & Bray, R. A. *Keys to the Trematoda: Volume 1*. (CABI publishing, 2002).

14 Thieltges, D. W. *et al.* Production of marine trematode cercariae: a potentially overlooked path of energy flow in benthic systems. *Mar. Ecol. Prog.* **372**, 147-155 (2008).

15 Brooks, M. E. *et al.* glmmTMB balances speed and flexibility among packages for zero-inflated generalized linear mixed modeling. *R Journal* **9**, 378-400 (2017).

16 Poulin, R. Macroecological patterns of species richness in parasite assemblages. *Basic Appl. Ecol.* **5**, 423-434 (2004).

17 Fenton, A., Viney, M. E. & Lello, J. Detecting interspecific macroparasite interactions from ecological data: patterns and process. *Ecol. Lett.* **13**, 606-615 (2010).

18 Poulin, R. *Evolutionary Ecology of Parasites*. (Princeton University Press, 2007).

19 Bolker, B. M. *et al.* Generalized linear mixed models: a practical guide for ecology and evolution. *Trends in Ecol. Evol.* **24**, 127-135 (2009).

20 Zuur, A. F., Ieno, E. N., Walker, N. J., Saveliev, A. A. & Smith, G. M. *Mixed Effects Models and Extensions in Ecology with R*. Vol. 574 (Springer, 2009).

21 Magnusson, M., Fischhoff, I. R., Ecke, F., Hörnfeldt, B. & Ostfeld, R. S. Effect of spatial scale and latitude on diversity–disease relationships. *Ecol.* **101**, e02955 (2020).
22 Halliday, F. W. & Rohr, J. R. Measuring the shape of the biodiversity-disease relationship across systems reveals new findings and key gaps. *Nat. Commun.* **10**, 5032 (2019).
23 Fox, J. *et al. Package 'car'*, <https://cran.r-project.org/web/packages/car/car.pdf> (2020).
24 Stewart Merrill, T. E. *et al.* Timescale reverses the relationship between host density and infection risk. *Proc. Roy. Soc. B* **289**, 20221106 (2022).
25 R Core Team. *R: A language and environment for statistical computing*, <https://www.R-project.org/> (2022).
26 Nieuwenhuis, R., Te Grotenhuis, H. & Pelzer, B. Influence.ME: tools for detecting influential data in mixed effects models. *R Journal* **4**, 38-47 (2022).
27 Lüdecke, D., Ben-Shachar, M. S., Patil, I., Waggoner, P. & Makowski, D. Performance: an R package for assessment, comparison and testing of statistical models. *JOSS* **6**, 3139-3149 (2021).
28 Stewart Merrill, T. E., Calhoun, D. M. & Johnson, P. T. J. Beyond single host, single parasite interactions: Quantifying competence for complete multi-host, multi-parasite communities. *Funct. Ecol.* **36**, 1845-1857 (2022).

Table S1. Model results for infection success at the individual host scale. For each parasite, we present estimates, standard errors, z-statistics and *P*-values for each predictor. Estimates come from a generalized linear mixed model (GLMM) with an overdispersed Poisson distribution that includes terms for infection pressure ($\log_{10}$ + 1 transformed), host richness, focal host density ($\log_{10}$ + 1 transformed density of *P. regilla*), predator density ($\log_{10}$ + 1 transformed density of damselfly larvae), and two-way interaction terms between infection pressure and each of the other terms. All numeric predictors were mean-centered and scaled (divided by 1 SD) prior to inclusion. The response variable is the number of trematode metacercariae within focal hosts (*Pseudacris regilla*). Only sites at which a parasite species was detected (in amphibians or snails) were included in the analysis, thereby focusing on factors that alter transmission between snails and amphibians, rather than on colonization to a site. Sample sizes, conditional (R^{2c}) and marginal (R^{2m}) *R*-squared values for each model are shown under the parasite name in the first column. Significant predictors ($P < 0.05$) are shown in bold.

Parasite	Predictor	Estimate	SE	z	*P*
Alaria	**Intercept**	**-4.085**	**0.342**	**-11.931**	**<0.00001**
N = 346	**Pressure**	**3.312**	**0.251**	**13.186**	**<0.00001**
R^{2c} = 0.41	Richness	0.0678	0.234	0.290	0.772
R^{2m} = 0.37	Density	0.0945	0.228	0.415	0.678
	Predators	-0.1059	0.229	-0.461	0.6446
	**Pressure x richness**	**-0.3937**	**0.1877**	**-2.098**	**0.0359**
	Pressure x density	0.1218	0.1884	0.647	0.5178
	Pressure x predators	-0.2267	0.1766	-1.284	0.1993
Cephalogonimus	**Intercept**	**-4.548**	**0.407**	**-11.190**	**<0.00001**
N = 495	**Pressure**	**2.247**	**0.1885**	**11.923**	**<0.00001**
R^{2c} = 0.50	Richness	0.0539	0.193	0.279	0.780
R^{2m} = 0.26	**Density**	**0.488**	**0.194**	**2.514**	**0.0119**
	Predators	0.0059	0.191	0.031	0.975
	**Pressure x richness**	**-0.374**	**0.157**	**-2.379**	**0.0174**
	Pressure x density	-0.132	0.142	-0.930	0.352
	Pressure x predators	0.1703	0.143	1.191	0.234
Echinostoma	**Intercept**	**2.033**	**0.190**	**10.679**	**<0.00001**
N = 432	**Pressure**	**0.964**	**0.101**	**9.537**	**<0.00001**
R^{2c} = 0.45	Richness	0.157	0.113	1.384	0.166
R^{2m} = 0.20	Density	0.0988	0.112	0.882	0.378
	Predators	-0.181	0.102	-1.770	0.0767
	**Pressure x richness**	**-0.320**	**0.100**	**-3.193**	**0.0014**
	Pressure x density	0.0078	0.089	0.087	0.930
	Pressure x predators	0.102	0.0986	1.0322	0.3022
Ribeiroia	**Intercept**	**-1.883**	**0.299**	**-6.285**	**<0.00001**
N = 496	**Pressure**	**2.184**	**0.166**	**13.123**	**<0.00001**
R^{2c} = 0.39	Richness	0.231	0.147	1.567	0.117
R^{2m} = 0.29	Density	-0.0692	0.1529	-0.452	0.651
	Predators	-0.074	0.143	-0.529	0.597
	**Pressure x richness**	**-0.711**	**0.137**	**-5.195**	**<0.00001**
	**Pressure x density**	**0.251**	**0.121**	**2.079**	**0.0376**
	Pressure x predators	0.174	0.131	1.330	0.184

Table S2. Model results for infection success at the individual scale for all five host species. Here, we evaluated how infection pressure, host species identity, and host richness affected parasite infection success within individual amphibian hosts. For each parasite, we present an analysis of deviance with the influence of each variable or interaction based on a χ^2 analysis comparing the model with that term to a reduced model without it. The χ^2 value, degrees of freedom, and *P*-value for each term are listed in the table. For the model of *Alaria* infection, the two newt species (*Taricha* spp.) were removed from the analysis owing to a near complete absence of infection. Significant predictors ($P < 0.05$) are shown in bold and sample sizes are provided below the parasite name in the first column.

**Parasite**	**Predictor**	χ^2	**df**	***P***
Alaria	**Pressure**	**171.24**	**1**	**<0.00001**
N = 478	Richness	0.008	1	0.931
	**Host species**	**42.38**	**2**	**<0.00001**
	**Pressure x richness**	**4.895**	**1**	**0.0269**
	Pressure x richness x host species	3.763	2	0.1524
Cephalogonimus	**Pressure**	**144.657**	**1**	**<0.00001**
N = 1088	Richness	0.163	1	0.686
	**Host species**	**145.228**	**4**	**<0.00001**
	**Pressure x richness**	**9.515**	**1**	**0.00204**
	Pressure x richness x host species	2.706	4	0.608
Echinostoma spp.	**Pressure**	**67.515**	**1**	**<0.00001**
N = 1079	Richness	1.457	1	0.227
	**Host species**	**970.932**	**4**	**<0.00001**
	**Pressure x richness**	**4.739**	**1**	**0.0294**
	Pressure x richness x host species	6.008	4	0.1985
Ribeiroia	**Pressure**	**187.87**	**1**	**<0.00001**
N = 1091	Richness	2.79	1	0.0945
	**Host species**	**99.56**	**4**	**<0.00001**
	**Pressure x richness**	**28.451**	**1**	**<0.00001**
	**Pressure x richness x host species**	**12.699**	**4**	**0.0128**

Table S3. Model results for infection success at the individual scale for all five host species. Results derived from the same models reported in Table S2, but while that table displays overall effects of predictors (generated with 'car::anova' in R), here we report the effects of each host species (generated with 'summary' in R). Models predict the total number of parasites in each species in a pond (with an offset for number of individuals examined to convert to parasites per host) and include a categorical variable identifying the relevant amphibian species identity. Fixed effects included a term for amphibian species identity, host richness, infection pressure, a pairwise interaction between infection pressure and host richness, and a three-way interaction between infection pressure, host richness, and amphibian species identity. All numeric predictors were mean-centered and scaled (divided by 1 SD) prior to inclusion. Populations of different host species were nested within ponds using a random intercept term. The three-way interaction effect was removed from models in which it was not significant (based on an analysis of deviance, see Table S2). For the model involving *Alaria*, the two newt species (TAGR and TATO) were removed because they are not susceptible to infection and including them lead to model failure. Species codes derived from first two letters of genus and of species: PSRE = *Pseudacris regilla*, RACA = *Rana catesbeiana*, TAGR = *Taricha granulosa*, TATO = *Taricha torosa*. The final species, *Anaxyrus boreas*, is the reference species in models. Marginal R^{2m} and conditional R^{2c} values are presented in the first column, along with sample size (*N*) for each parasite. Significant predictors ($P < 0.05$) are shown in bold.

Parasite	Predictor	Estimate	SE	z	*P*
Alaria	**Intercept**	**-6.7142**	**0.5489**	**-12.231**	**<0.00001**
N = 478	**Pressure**	**3.1241**	**0.2452**	**12.739**	**<0.00001**
R^{2c} = 0.57	Richness	0.1046	0.2325	0.450	0.6528
R^{2m} = 0.36	**PSRE**	**2.7365**	**0.4519**	**6.055**	**<0.00001**
	RACA	0.2259	0.8508	0.266	0.7906
	**Pressure x richness**	**-0.4233**	**0.1954**	**-2.166**	**0.030**
Cephalogonimus	**Intercept**	**-7.0770**	**0.5878**	**-12.040**	**<0.00001**
N = 1088	**Pressure**	**2.2676**	**0.1895**	**11.968**	**<0.00001**
R^{2c} = 0.52	Richness	0.0786	0.1910	0.411	0.68073
R^{2m} = 0.36	**PSRE**	**2.1004**	**0.4638**	**4.528**	**<0.00001**
	RACA	0.2468	1.1654	0.212	0.8323
	**TAGR**	**-2.6749**	**0.8182**	**-3.269**	**0.0011**
	**TATO**	**-3.5639**	**0.6421**	**-5.551**	**<0.00001**
	**Pressure x richness**	**-0.4560**	**0.1431**	**-3.186**	**0.0014**
Echinostoma spp.	Intercept	0.02278	0.2787	0.082	0.9349
N = 1079	**Pressure**	**0.8813**	**0.1099**	**8.016**	**<0.00001**
R^{2c} = 0.67	Richness	0.1555	0.1185	1.312	0.1896
R^{2m} = 0.52	**PSRE**	**1.9419**	**0.2562**	**7.581**	**<0.00001**
	**RACA**	**2.111**	**0.4761**	**4.435**	**<0.00001**
	**TAGR**	**-4.8496**	**0.4175**	**-11.617**	**<0.00001**
	**TATO**	**-4.3840**	**0.2956**	**-14.709**	**<0.00001**
	**Pressure x richness**	**-0.2201**	**0.1011**	**-2.176**	**<0.00001**
Ribeiroia	**Intercept**	**-3.3664**	**0.3422**	**-0.9839**	**<0.00001**
N = 1091	**Pressure**	**2.1188**	**0.1448**	**14.633**	**<0.00001**
R^{2c} = 0.67	Richness	0.2334	0.1255	1.860	0.0628
R^{2m} = 0.29	**PSRE**	**1.5168**	**0.2126**	**7.133**	**<0.00001**
	**RACA**	**1.9782**	**0.3978**	**4.973**	**<0.00001**
	**TAGR**	**1.0663**	**0.3081**	**3.461**	**<0.00001**
	**TATO**	**2.0550**	**0.2280**	**9.012**	**<0.00001**

	**Pressure x richness**	**-0.6742**	**0.1566**	**-4.304**	**<0.00001**
	Pressure x richness x PSRE	0.0144	0.1275	0.113	0.9100
	**Pressure x richness x RACA**	**-0.3740**	**0.1745**	**-2.143**	**0.0321**
	Pressure x richness x TAGR	0.3690	0.2276	1.622	0.1048
	Pressure x richness x TATO	0.1642	0.1358	1.208	0.2269

Table S4. Model results for infection success at the host community scale. For each parasite, we present estimates, standard errors, z-statistics and *P*-values for each predictor coefficient retained in the final model. Estimates come from a linear mixed model (LMM) with a Gaussian distribution and terms for infection pressure, host richness, total host density (summed among all host species in the same pond), predator density, and two-way interaction terms between infection pressure and each of the other terms. All numeric predictors were mean-centered and scaled (divided by 1 SD) prior to inclusion. The response variable is the density of trematode metacercariae (number per dipnet) within all amphibian hosts within the same community (log_{10}+1 transformed). Only sites at which a parasite species were detected (in amphibians or snails) and in which all non-endangered hosts were sampled were included in the analysis. Marginal R^{2m} and conditional R^{2c} values are presented in the first column, along with sample size (*N*) for each parasite. The model for *Alaria* omits a random intercept term for pond identity owing to singularity. Significant predictors ($P < 0.05$) are shown in bold.

Parasite	Predictor	Estimate	SE	z	*P*
Alaria	**Intercept**	**0.2923**	**0.0432**	**6.770**	**<0.00001**
N = 175	**Pressure**	**0.3420**	**0.0371**	**9.230**	**<0.00001**
R^{2c} = 0.39	Richness	-0.0155	0.0369	-0.422	0.6729
R^{2m} = 0.38	**Density**	**0.1454**	**0.0391**	**3.717**	**0.0002**
	Predators	-0.0051	0.0367	-0.140	0.8889
	**Pressure x richness**	**-0.0886**	**0.0341**	**-2.596**	**0.0094**
	**Pressure x density**	**0.1584**	**0.0417**	**3.797**	**0.00015**
	Pressure x predators	-0.0397	0.0369	-1.074	0.2828
Cephalogonimus	**Intercept**	**0.2062**	**0.0335**	**6.166**	**<0.00001**
N = 250	**Pressure**	**0.2476**	**0.0243**	**10.183**	**<0.00001**
R^{2c} = 0.69	Richness	-0.0302	0.0240	-1.255	0.2095
R^{2m} = 0.65	**Density**	**0.1883**	**0.0235**	**7.996**	**<0.00001**
	**Predators**	**0.0466**	**0.0222**	**2.098**	**0.0359**
	**Pressure x richness**	**-0.0715**	**0.0238**	**-3.007**	**0.0026**
	**Pressure x density**	**0.2669**	**0.0254**	**10.503**	**<0.00001**
	**Pressure x predators**	**0.0414**	**0.0210**	**1.969**	**0.049**
Echinostoma spp.	**Intercept**	**1.206**	**0.0652**	**18.507**	**<0.00001**
N = 259	**Pressure**	**0.2881**	**0.0421**	**6.831**	**<0.00001**
R^{2c} = 0.59	Richness	0.0012	0.0471	0.026	0.979
R^{2m} = 0.43	**Density**	**0.4849**	**0.0465**	**10.430**	**<0.00001**
	Predators	-0.0421	0.0435	-0.966	0.334
	Pressure x richness	-0.0626	0.0419	-1.493	0.135
	Pressure x density	0.0251	0.0398	0.630	0.529
	Pressure x predators	0.0191	0.0423	0.453	0.651
Ribeiroia	**Intercept**	**0.5595**	**0.0685**	**8.161**	**<0.00001**
N = 252	**Pressure**	**0.5027**	**0.0372**	**13.502**	**<0.00001**
R^{2c} = 0.62	Richness	0.0247	0.0351	0.704	0.482
R^{2m} = 0.52	**Density**	**0.1173**	**0.0336**	**3.485**	**0.0005**
	Predators	-0.0205	0.0312	-0.659	0.510
	**Pressure x richness**	**-0.1082**	**0.0282**	**-3.829**	**0.00013**
	**Pressure x density**	**0.1410**	**0.0318**	**4.429**	**<0.00001**
	Pressure x predators	-0.0057	0.0300	-0.192	0.848

Table S5. Model results for infection success at the community scale using community competence as the explanatory variable. As in Table S4, the response variable is the density of trematode metacercariae (number per dipnet) within all amphibian hosts within the same community (log_{10}+1 transformed). As an alternative to using specific terms for host richness and host density, we constructed models with community competence and its interaction with infection pressure. Community competence represents the density-weighted values of each amphibian species' lab-derived competence value, thereby incorporating information on host identity, host density, and host suitability to infection by a specific parasite. For each parasite, we present estimates, standard errors, z-statistics and *P*-values for each coefficient. All numeric predictors were mean-centered and scaled (divided by 1 SD) prior to inclusion. Delta AIC values (far left column) refer to comparisons against a corresponding model that also included infection pressure but, instead of competence, included host richness, total host density (summed among all host species in the same pond), and two-way interactions between these terms and infection pressure (note that predator density is not included in either model). Negative values indicate lower AICs (and hence greater support) for the competence model formulation. Conditional and marginal R^2 values of the competence models are also presented in the first column, along with sample size for each parasite. Significant predictors (P < 0.05) are shown in bold.

Parasite	Predictor	Estimate	SE	z	*P*
Alaria	**Intercept**	**0.2897**	**0.0352**	**8.233**	**<0.00001**
N = 175	**Pressure**	**0.3585**	**0.0343**	**10.446**	**<0.00001**
ΔAIC = -25<br>R^{2c} = 0.46<br>R^{2m} = 0.45	**Competence**	**0.1739**	**0.0354**	**4.921**	**<0.00001**
	**Pressure x competence**	**0.1947**	**0.0366**	**5.327**	**<0.00001**
Cephalogonimus	**Intercept**	**0.2154**	**0.0267**	**8.068**	**<0.00001**
N = 250	**Pressure**	**0.2753**	**0.0231**	**11.902**	**<0.00001**
ΔAIC = -16.3<br>R^{2c} = 0.69<br>R^{2m} = 0.65	**Competence**	**0.1799**	**0.0220**	**8.169**	**<0.00001**
	**Pressure x competence**	**0.2419**	**0.0219**	**11.014**	**<0.00001**
Echinostoma	**Intercept**	**1.193**	**0.0625**	**19.099**	**<0.00001**
N = 259	**Pressure**	**0.309**	**0.0405**	**7.636**	**<0.00001**
ΔAIC = -19.3<br>R^{2c} = 0.60<br>R^{2m} = 0.46	**Competence**	**0.517**	**0.0437**	**11.833**	**<0.00001**
	Pressure x competence	0.07133	0.0396	1.803	0.0714
Ribeiroia	**Intercept**	**0.5331**	**0.0668**	**7.980**	**<0.00001**
N = 252	**Pressure**	**0.433**	**0.0319**	**13.595**	**<0.00001**
ΔAIC = +5.8<br>R^{2c} = 0.61<br>R^{2m} = 0.50	**Competence**	**0.1393**	**0.0336**	**4.148**	**<0.00001**
	**Pressure x competence**	**0.1128**	**0.0296**	**3.802**	**0.00014**

Table S6. Relationship between snail size (in mm) and the number of released cercariae for each parasite taxon. Snails were collected from field sites and kept isolated in 50 mm centrifuge tubes filled with approximately 40 ml of artificial spring water for 24 hours. Tubes were checked for the presence of cercariae at multiple time points (morning and afternoon), and cercariae were counted with the aid of a dissecting microscope. Using linear regression analysis, we determined how the number of cercariae released ($\log_{10}$-transformed + 1) related to snail size. Thus, deriving the estimated number of cercariae per snail is as follows: 10^(intercept + snail size x slope coefficient).

Trematode	Snail	Intercept	Slope for size	R^2	*N*
Strigeid (*Alaria*)	*H. trivolvis*	1.442 ± 0.199	0.061 ± 0.015**	0.04	387
Armatae (*Cephalogonimus*)	*H. trivolvis*	1.172 ± 0.378	0.059 ± 0.026*	0.04	113
Echinostoma	*H. trivolvis*	0.012 ± 0.286	0.131 ± 0.018***	0.26	140
Echinostoma	*Physa* sp.	1.045 ± 0.298	0.029 ± 0.0296	0.06	19
Ribeiroia	*H. trivolvis*	0.175 ± 0.104	0.110 ± 0.006***	0.22	1041

Table S7. To estimate infection pressure, we compared three proxies: infection prevalence in snails, density of infected snails, and estimated density of infective cercariae. We evaluated which proxy best represented exposure by regressing each against infection success (metacercariae per host) in *P. regilla* (our focal species) and comparing model fit with AIC. For each parasite (leftmost column), columns show the AIC value for each proxy, followed by a column noting the best fitting model (that with the lowest AIC value, also indicated in bold) and a ΔAIC value that indicates by how many units the best model performed relative the next best model (i.e., for *Alaria*, the model for cercaria density was 159 AIC units lower than that for infected snail density).

**Parasite**	**Prevalence in snails**	**Infected snail density**	**Cercaria density**	**Best fitting model**	**ΔAIC**
Alaria	1806	1795	**1636**	**Cercaria density**	-159
Cephalogonimus	1990	1996	**1874**	**Cercaria density**	-116
Echinostoma	5614	5641	**5607**	**Cercaria density**	-7
Ribeiroia	3347	3354	**3249**	**Cercaria density**	-98

Table S8. Correlation matrices showing Pearson *r* values for the fixed effects in transmission models for each parasite at the individual and community scales. While host richness, host density, and predator density were not collinear (all |*r*| < 0.3), there were strong positive correlations between total host density and community competence (all *r* > 0.8; indicated in red). This was not surprising given that community competence is a competence-weighted value of total host density (and therefore includes density in the calculation). Because of this collinearity, we ran two separate models at the community scale: one that incorporated infection pressure, host richness, total host density and predator density and a model that included infection pressure and community competence.

Alaria – individual scale

	**Cercaria density**	**Host richness**	**Host density**	**Predator density**
**Cercaria density**				
**Host richness**	0.038			
**Host density**	0.018	-0.001		
**Predator density**	0.161	0.004	-0.169	

Cephalogonimus – individual scale

	**Cercaria density**	**Host richness**	**Host density**	**Predator density**
**Cercaria density**				
**Host richness**	0.026			
**Host density**	0.1381	0.037		
**Predator density**	0.076	0.012	-0.119	

Echinostoma – individual scale

	**Cercaria density**	**Host richness**	**Host density**	**Predator density**
**Cercaria density**				
**Host richness**	0.144			
**Host density**	0.139	0.042		
**Predator density**	0.110	0.060	-0.101	

Ribeiroia – individual scale

	**Cercaria density**	**Host richness**	**Host density**	**Predator density**
**Cercaria density**				
**Host richness**	0.216			
**Host density**	0.040	0.038		
**Predator density**	-0.022	0.014	-0.123	

Alaria – community scale

	**Cercaria density**	**Host richness**	**Total host density**	**Predator density**	**Community competence**
**Cercaria density**					
**Host richness**	0.038				
**Total host density**	-0.049	0.159			
**Predator density**	0.160	-0.044	-0.284		
**Community competence**	-0.054	-0.067	**0.847**	-0.205	

Cephalogonimus – community scale

	**Cercaria density**	**Host richness**	**Total host density**	**Predator density**	**Community competence**
**Cercaria density**					
**Host richness**	0.032				
**Total host density**	0.153	0.159			
**Predator density**	0.078	-0.019	-0.213		

**Community competence**	0.147	0.034	**0.829**	-0.172	

Echinostoma – community scale

	Cercaria density	Host richness	Total host density	Predator density	Community competence
**Cercaria density**					
**Host richness**	0.092				
**Total host density**	0.067	0.154			
**Predator density**	0.112	0.043	-0.201		
**Community competence**	0.010	0.038	**0.829**	-0.149	

Ribeiroia – community scale

	Cercaria density	Host richness	Total host density	Predator density	Community competence
**Cercaria density**					
**Host richness**	0.232				
**Total host density**	0.129	0.164			
**Predator density**	-0.061	-0.019	-0.216		
**Community competence**	0.149	0.065	**0.928**	-0.168	

Table S9. Mean infection loads (metacercariae per host) and standard errors for each trematode species within each non-endangered amphibian species. Values are calculated from site-year combinations in which both the host and parasite are present (as indicated by positive infections either in snails or any amphibian species from the site). Mean values are derived from average infection load per site-year in a given species, for which uninfected hosts are included. The number of site-years is presented in the final column.

Alaria	**Mean infection**	**SE**	***N***
A. boreas	1.54	1.02	105
P. regilla	4.82	0.976	346
R. catesbeiana	0.754	0.644	27
T. granulosa	0	0	69
T. torosa	0.0058	0.0048	210
Cephalogonimus			
A. boreas	0.628	0.353	143
P. regilla	1.98	0.351	495
R. catesbeiana	0.137	0.134	35
T. granulosa	0.383	0.263	108
T. torosa	0.021	0.0074	307
Echinostoma			
A. boreas	12.9	2.49	136
P. regilla	38.2	3.16	432
R. catesbeiana	123.0	29.2	37
T. granulosa	1.04	0.652	122
T. torosa	1.55	0.39	352
Ribeiroia			
A. boreas	1.64	0.385	144
P. regilla	4.17	0.489	496
R. catesbeiana	10.9	2.59	35
T. granulosa	6.77	5.33	108
T. torosa	7.50	1.33	308

Table S10. Scaled competence values (rounded to two decimal places) for the five amphibian hosts (rows) and four trematode parasites (columns). Note: some host-parasite interactions had very low but non-zero scaled competence values that appear as zeros when rounded. Absolute competence values are provided in Stewart Merrill *et al.* (2022), *Functional Ecology*.

	Alaria	***Cephalogonimus***	***Echinostoma***	***Ribeiroia***
A. boreas	0.37	1.00	0.59	0.00
P. regilla	1.00	0.67	0.70	0.46
R. catesbeiana	0.20	0.92	1.00	1.00
T. granulosa	0.00	0.00	0.00	0.12
T. torosa	0.00	0.00	0.00	0.35

Figure S1. Regression plots for assessing the best metric for 'infection pressure'. To estimate infection pressure, we calculated infection prevalence in snails, density of infected snails, and estimated density of infective cercariae, then evaluated which proxy term best fit data on parasite infection success for each trematode parasite. The resulting regressions (below) consist of each proxy on the x-axis and infection success (metacercariae per host) in *P. regilla* on the y-axis. Estimated cercaria densities (rightmost panels) led to the best model fits, as indicated by AIC (Table S7), and visual fits of the data.

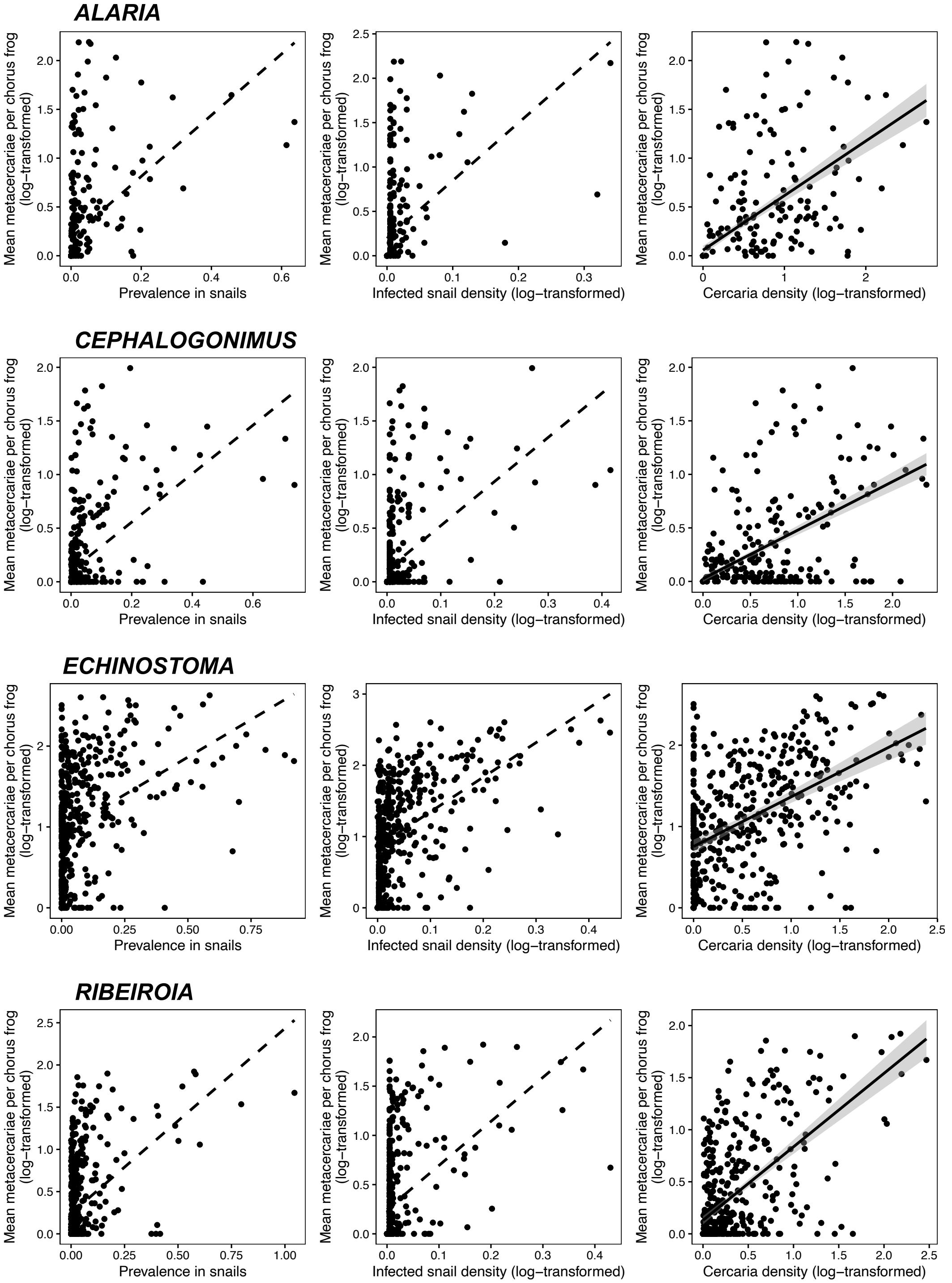

Figure S2. Relationship between host richness and total host density. Presented are two model forms (see 'Relationship between host density and host richness' in the supplement) illustrating the relationship between amphibian species richness (on the x-axis) and total number of amphibian larvae caught per dipnet sweep (summed among species) for all pond-year sampling events. The left panel displays the raw data while the right panel illustrates the same data but with a $\log_{10}+1$ transformation of amphibian density. The black line represents a linear fit to the data and the red dashed line represents a saturating fit, both of which yielded comparable model AIC values. This suggests a linear (additive) relationship between richness and density is adequate for these observations. See text for additional details.

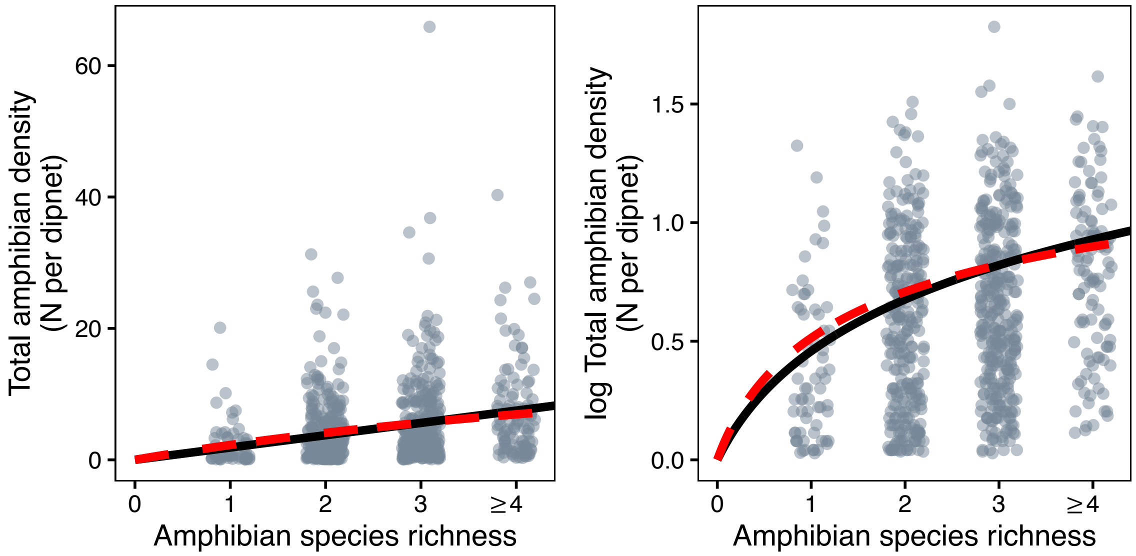

Supplement: Supplementary file 1 — Supplementary Information [file 41467_2024_46091_MOESM1_ESM.pdf]
