# Supplementary material for: Diverging effects of host density and richness across biological scales drive diversity-disease outcomes
Source: Nat Commun. 2024 Mar 2;15:1937. doi: 10.1038/s41467-024-46091-4 (PMC10908850; doi:10.1038/s41467-024-46091-4)
Supplement: Supplementary file 3 — Reporting Summary [file 41467_2024_46091_MOESM3_ESM.pdf]

nature portfolio

Corresponding author(s): Pieter Johnson

Last updated by author(s): Feb 5, 2024

Reporting Summary

Nature Portfolio wishes to improve the reproducibility of the work that we publish. This form provides structure for consistency and transparency in reporting. For further information on Nature Portfolio policies, see our Editorial Policies and the Editorial Policy Checklist.

Statistics

For all statistical analyses, confirm that the following items are present in the figure legend, table legend, main text, or Methods section.

n/a	Confirmed	
☐	☒	The exact sample size (*n*) for each experimental group/condition, given as a discrete number and unit of measurement
☐	☒	A statement on whether measurements were taken from distinct samples or whether the same sample was measured repeatedly
☐	☒	The statistical test(s) used AND whether they are one- or two-sided<br>*Only common tests should be described solely by name; describe more complex techniques in the Methods section.*
☐	☒	A description of all covariates tested
☐	☒	A description of any assumptions or corrections, such as tests of normality and adjustment for multiple comparisons
☐	☒	A full description of the statistical parameters including central tendency (e.g. means) or other basic estimates (e.g. regression coefficient) AND variation (e.g. standard deviation) or associated estimates of uncertainty (e.g. confidence intervals)
☐	☒	For null hypothesis testing, the test statistic (e.g. *F*, *t*, *r*) with confidence intervals, effect sizes, degrees of freedom and *P* value noted<br>*Give P values as exact values whenever suitable.*
☒	☐	For Bayesian analysis, information on the choice of priors and Markov chain Monte Carlo settings
☐	☒	For hierarchical and complex designs, identification of the appropriate level for tests and full reporting of outcomes
☐	☒	Estimates of effect sizes (e.g. Cohen's *d*, Pearson's *r*), indicating how they were calculated

Our web collection on statistics for biologists contains articles on many of the points above.

Software and code

Policy information about availability of computer code

Data collection	No software was used
Data analysis	All analyses were conducted in R version 4.3.1 (R Core Team 2023)

For manuscripts utilizing custom algorithms or software that are central to the research but not yet described in published literature, software must be made available to editors and reviewers. We strongly encourage code deposition in a community repository (e.g. GitHub). See the Nature Portfolio guidelines for submitting code & software for further information.

Data

Policy information about availability of data

All manuscripts must include a data availability statement. This statement should provide the following information, where applicable:

- Accession codes, unique identifiers, or web links for publicly available datasets
- A description of any restrictions on data availability
- For clinical datasets or third party data, please ensure that the statement adheres to our policy

The data generated in this study have been deposited in the Figshare database under DOI: 10.6084/m9.figshare.24982794. Source data are provided with this paper.

Research involving human participants, their data, or biological material

Policy information about studies with human participants or human data. See also policy information about sex, gender (identity/presentation), and sexual orientation and race, ethnicity and racism.

Reporting on sex and gender	N/A
Reporting on race, ethnicity, or other socially relevant groupings	N/A
Population characteristics	N/A
Recruitment	N/A
Ethics oversight	N/A

Note that full information on the approval of the study protocol must also be provided in the manuscript.

Field-specific reporting

Please select the one below that is the best fit for your research. If you are not sure, read the appropriate sections before making your selection.

☐ Life sciences ☐ Behavioural & social sciences ☒ Ecological, evolutionary & environmental sciences

For a reference copy of the document with all sections, see nature.com/documents/nr-reporting-summary-flat.pdf

Ecological, evolutionary & environmental sciences study design

All studies must disclose on these points even when the disclosure is negative.

Study description	We used data from interactions between amphibian hosts and trematode parasites to test how changes in host density, host community richness, and predators altered parasite transmission. The structure of the data was hierarchical. Parasite infection load was quantified within individual hosts and also summed among individuals and species to estimate the population-level abundance of a parasite. Individual hosts (n=17,337) and populations occurred within ponds (n=224), which were sampled across multiple parks/ metacommunities (n=8) and through time (11 years). Field surveys were complemented with experimental studies to measure each amphibian host species' competence for supporting infection by each trematode species.
Research sample	The research sample included larval and metamorphic amphibians from lentic habitats in the East Bay region of California. This is a useful area in which to research because the species composition is tractable and there are numerous replicate communities that vary in key attributes, such as diversity. The primary species sampled were: Pseudacris regilla (Pacific chorus frog), Anayxrus boreas (western toad), Rana catesbeiana (American bullfrog), Taricha torosa (California newt), and T. granulosa (rough-skinned newt). Two additional species were quantified in the field but not processed for parasite infections. These were Rana draytonii (California red-legged frog) and Ambystoma californiense (California tiger salamander). Both species are threatened at the state or federal level. The age range of sampled species were from larval stages (1 week to 1 year in age, depending on the species) and recently metamorphosed individuals (approximately 2 months to 1.5 years in age, depending on the species). For field surveys, amphibian hosts were either sampled and released or, for a subset, processed to quantify naturally occurring parasite infections. For experimental infections, egg masses or early stage larvae of each amphibian species were collected from field sites and subsequently exposed to one of four trematode taxa (Ribeiroia, Alaria, Cephalogonimus, Echinostoma) and one of five ecologically-relevant exposure dosages (0 [control], 20, 40, 100, or 200 cercariae). In addition to amphibians, the study also quantified the density and trematode infection prevalence patterns within freshwater snails, including Helisoma spp. and Physa spp.
Sampling strategy	Sampling was performed at field sites using a range of techniques, such as dipnet samples, seining, visual encounter surveys, and hand capture. Sample sizes were selected for field surveys and experiments based on previous studies, tractability, and system experience. We used statistical techniques such as GLMMs that can handle unbalanced designs and rely on partial pooling to improve coefficient estimation
Data collection	Data were collected at field sites using dipnet samples, seining, visual encounter surveys, and hand capture. For field data and necropsies, counts of observed taxa, host attributes, and environmental data were recorded using pencil and waterproof paper. Individuals in charge of data collection included the following. Field data collection: Daniel Preston, Katherine Richgels, Sara Paull, Travis McDevitt-Galles, Wynne Moss, Brendan Hobart, Ty McCaffrey, Dylan Rose, Pieter Johnson. Laboratory data collection: Dana Calhoun, Jessica Quinn, Tawni Riepe, Katie Leslie, Bryan LaFonte, Erica Ursich, Sarah Orlofske, Pieter Johnson.
Timing and spatial scale	Sampling was performed annually between 2009 and 2019. Visits began in April/May of each year and ended in August. Typically field sites were visited 2 to 3x per year, for which the rationale was to provide an early season visit (focused on amphibian larvae) and a later season visit (focused on metamorphosing amphibians, which offer a standardized stage in which to assess infection). Sampling was generally conducted during daylight hours, with the exception of night-time transects for bullfrogs (which increases capture success).Individual ponds vary in size from approximately 10 m in perimeter to 1000 m depending on precipitation in a given year, and are distributed across a three county area of California (approximately 713,000 ha).

Data exclusions

The presence and potential influence of outliers were explored using three methods. First, we generated plots of the relationship between infection pressure (from snail intermediate hosts) and observed amphibian infection loads for each trematode, with estimated cercariae density on the x-axis and mean metacercariae per chorus frog on the y-axis (both variables were logl0 + 1 transformed). Any observations for which pressure was high (>1.5) and amphibian infection approached zero were considered potential outliers and further examined or removed. Second, we investigated residuals from the fitted relationship between infection pressure and amphibian parasite load, for which potential outliers were considered as observations whose residuals were more than 3 standard deviations greater than the mean residual. Third, we calculated Cook's distance from our standard model (using the package influence.ME), where potential outliers were considered those whose distance was greater than 4/N. Any observations that were identified as potential outliers using at least two of the three methods were removed.

Reproducibility

Field work and experiments were completed across multiple years, for which we accounted for the effects of year or sampling block. Patterns were found to be robust and reproducible. This included 11 years of surveys for the field data (2009 to 2019). Experimental results were also compared to previously published studies (e.g., Johnson et al. 2012) and found to be consistent.

Randomization

For experimental studies, larval amphibians were randomly assigned to treatment for parasite exposure (dose as well as parasite identity). For field studies, hosts for dissection were selected haphazardly to create a representative sample.

Blinding

For experimental studies, laboratory personnel quantifying parasite loads were unaware of the treatment for each host, which were given unique identifying codes.

Did the study involve field work? ☒ Yes ☐ No

Field work, collection and transport

Field conditions

This region in California has a Mediterranean climate with dry, hot summers.

Location

Field surveys were conducted in the East Bay Region of California, including the counties of Santa Clara, Contra Costa and Alameda. Study sites include a large number of ponds and small lakes, typically under 2 ha in surface area, which were subjected to mixed use (recreation, livestock, wildlife conservation, municipal water storage).

Access & import/export

Permits were in compliance with local land managers (California State Parks, East Bay Regional Parks District, East Bay Municipal Utilities District, Santa Clara County Parks, Open Space Authority, Midpeninsula Open Space), state wildlife regulations (California Department of Fish and Wildlife), federal regulations (US Fish and Wildlife Service), and University of Colorado guidelines on use and care of animals (IACUC).

Disturbance

Field crew took care to avoid excessive trampling at field sites or disturbance of water in aquatic systems. Visits to sites were generally also limited in number (two to three visits) per summer. Because many sites have livestock use, the added influence of occasional visits by field crews was deemed to be small and in compliance with land manager regulations.

Reporting for specific materials, systems and methods

We require information from authors about some types of materials, experimental systems and methods used in many studies. Here, indicate whether each material, system or method listed is relevant to your study. If you are not sure if a list item applies to your research, read the appropriate section before selecting a response.

Materials & experimental systems

n/a	Involved in the study	
☒	☐	Antibodies
☒	☐	Eukaryotic cell lines
☒	☐	Palaeontology and archaeology
☐	☒	Animals and other organisms
☒	☐	Clinical data
☒	☐	Dual use research of concern
☒	☐	Plants

Methods

n/a	Involved in the study	
☒	☐	ChIP-seq
☒	☐	Flow cytometry
☒	☐	MRI-based neuroimaging

Animals and other research organisms

Policy information about studies involving animals; ARRIVE guidelines recommended for reporting animal research, and Sex and Gender in Research

Laboratory animals

We used larvae of non-threatened, lentic breeding amphibian species: Rana catesbeiana, Pseudacris regilla, Anaxyrus boreas, Taricha torosa, and Taricha granulosa. All were collected from field sites as eggs or early stage larvae.

Wild animals

We sampled larvae and metamorphs of lentic breeding amphibian species, specifically Rana draytonii, Ambystoma californiense,

Wild animals	Rana catesbeiana, Pseudacris regilla, Anaxyrus boreas, Taricha torosa, and Taricha granulosa. All were sampled at field sites in the East Bay Region of California, captured by hand or by net. All individuals of threatened species (i.e., R. draytonii and A. californiense) were immediately released following capture within the pond of origin. For non-threatened species, we released most individuals immediately following capture and collected a subset (~10 per species per site) to process for parasite infection. These individuals were humanely euthanized by exposure to pH buffered MS-222 (1 g/500 mL) and then necropsied to identify and quantify any associated parasites.
Reporting on sex	Sex was not considered as the study focused on immature stages
Field-collected samples	Any field-collected samples were processed shortly after collection, rather than maintained. For experimental studies, hatching larvae were maintained in carbon-filtered, UV-sterilized tapwater at 22 deg. C on a 12:12 hour photoperiod.
Ethics oversight	Permits were in compliance with local land managers {Santa Clara County Parks, East Bay Regional Park District, East Bay Municipal Utility District), state wildlife regulations (California Department of Fish and Wildlife), federal regulations (US Fish and Wildlife Service), and University of Colorado guidelines on use and care of animals (IACUC).

Note that full information on the approval of the study protocol must also be provided in the manuscript.

Plants

Seed stocks	N/A
Novel plant genotypes	N/A
Authentication	N/A
